# Carotenoids of Sea Angels *Clione limacina* and *Paedoclione doliiformis* from the Perspective of the Food Chain

**DOI:** 10.3390/md12031460

**Published:** 2014-03-13

**Authors:** Takashi Maoka, Takashi Kuwahara, Masanao Narita

**Affiliations:** 1 Research Institute for Production Development, Shimogamo-Morimoto-cho 15, Sakyo-ku, Kyoto 606-0805, Japan; 2 Okhotsk Sea Ice Museum of Hokkaido, Motomombetsu, Monbetsu, Hokkaido 094-0023, Japan; E-Mail: kanri1@giza-ryuhyo.com; 3 Hokkaido Research Organization, Abashiri Fisheries Research Institute, Minatomachi, Monbetsu, Hokkaido 094-0011, Japan; E-Mail: narita-masanao@hro.or.jp

**Keywords:** carotenoids, sea angels, food chain, metabolism

## Abstract

Sea angels, *Clione limacina* and *Paedoclione doliiformis*, are small, floating sea slugs belonging to Gastropoda, and their gonads are a bright orange-red color. Sea angels feed exclusively on a small herbivorous sea snail, *Limacina helicina*. Carotenoids in *C. limacina*, *P. doliiformis*, and *L. helicina* were investigated for comparative biochemical points of view. β-Carotene, zeaxanthin, and diatoxanthin were found to be major carotenoids in *L. helicina*. *L. helicina* accumulated dietary algal carotenoids without modification. On the other hand, keto-carotenoids, such as pectenolone, 7,8-didehydroastaxanthin, and adonixanthin were identified as major carotenoids in the sea angels *C. limacina* and *P. doliiformis*. Sea angels oxidatively metabolize dietary carotenoids and accumulate them in their gonads. Carotenoids in the gonads of sea angels might protect against oxidative stress and enhance reproduction.

## 1. Introduction

*Clione limacina* is a small, floating sea slug (0.5~3 cm body length) belonging to the family Clionidae, which is a group of pelagic marine gastropods. *Paedoclione doliiformis* is a very small, floating sea slug (<0.5 cm body length) that also belongs to the family Clionidae. Their shells are lost during development and their body is gelatinous and transparent. On the other hand, their gonads and viscera are a bright orange-red color. They float by flapping their “wings”. Their floating styles resemble angels and so they are called “sea angels” [1]. From spring to autumn, sea angels live at a depth of 200 m in the Sea of Okhotsk. In winter, they migrate to the coast of north Hokkaido with drift ice. The sea angels, *C. limacina* and *P. doliiformis*, are carnivorous and feed exclusively on *Limacina helicina*, which is a small, swimming predatory sea snail belonging to the family Limacinidae (Gastropoda) which feed on micro algae such as diatoms and dinoflagellates [2]. Chum salmon, *Oncorhynchus keta*, is one of the major predators of sea angels in the Okhotsk Sea of north Hokkaido [3,4].

Marine animals, especially marine invertebrates, contain various carotenoids, showing structural diversity [5,6,7,8]. New carotenoids are still being discovered in marine animals [9]. In general, animals do not synthesize carotenoids *de novo*, and so those found in animals are either directly accumulated from food or partly modified through metabolic reactions [6,7,8]. The major metabolic conversions of carotenoids found in marine animals are oxidation, reduction, the translation of double bonds, oxidative cleavage of double bonds, and cleavage of epoxy bonds. Therefore, structural diversity is found in carotenoids of marine animals [6,7,8].

**Figure 1:**
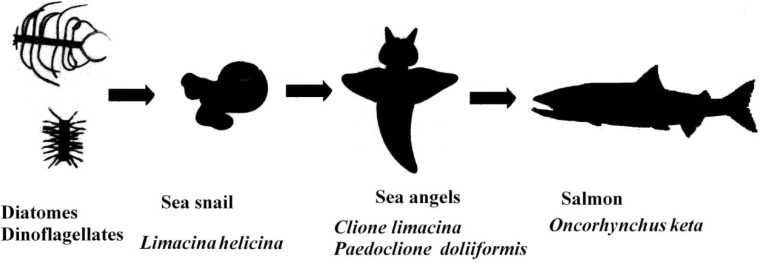
Food chains from phytoplankton to salmon via sea angels in the Okhotsk Sea of north Hokkaido.

We have studied carotenoids in several marine animals from chemical and comparative biochemical points of view [8,9,10]. We have been interested in the orange-red pigments, which were assumed to be carotenoids, of sea angels. Thus, we studied the carotenoids of the sea angels *C. limacina* and *P. doliiformis*. Furthermore, carotenoids in the small snail *L. helicina* and chum salmon *O. keta* were studied from the perspective of the food chain (Figure 1). In the present paper, we describe the carotenoids of these marine animals from the viewpoints of comparative biochemistry and the food chain.

## 2. Results

**Figure 2:**
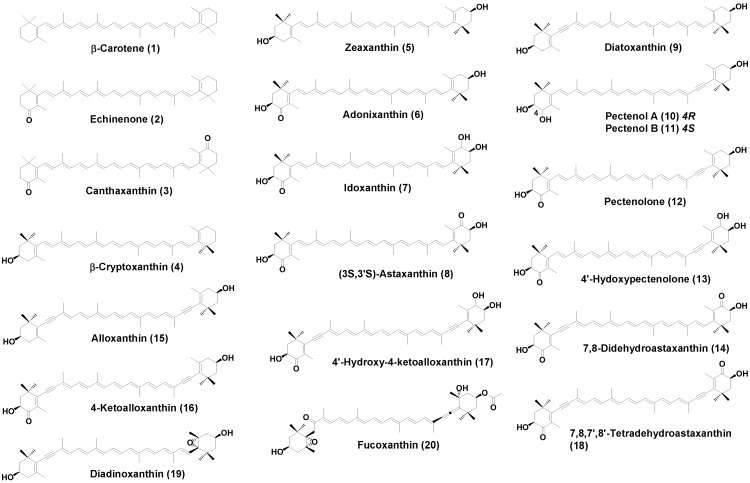
Structure of carotenoids found in *C. limacina*, *P. doliiformis*, and *L. helicina*.

Structural formulae of carotenoids identified from the sea angels *C. limacina* and *P. doliiformis* and the small herbivorous sea snail *L. helicina* are shown in Figure 2.

### 2.1. Carotenoids of L. helicina

The carotenoid content and composition of the small herbivorous sea snail *L. helicina* are shown in Table 1. The total carotenoid content of *L. helicina* was 21.0 μg/g wet weight. β-Carotene (32.2%), zeaxanthin (24.2%), diatoxanthin (11.1%), and β-cryptoxanthin (10.4%) were found to be major carotenoids. Characteristic algal carotenoids, fucoxanthin (5.2%) and diadinoxanthin (2.4%), were also found.

### 2.2. Carotenoids of C. limacinea

The carotenoid content and composition of the sea angel *C. limacina* are shown in Table 1. The total carotenoid content of *C. limacina* was 47.0 μg/g wet weight. Fifteen carotenoids were identified. β-Carotene (27.6%), β-cryptoxanthin (13.5%), and echinenone (9.2%) were found to be major components. Monoacetylenic carotenoids, such as diatoxanthin, 7,8-didehydroastaxanthin, pectenolone, pectenol A, pectenol B, and 4′-hydroxypectenolone, comprised 25.9% of the total carotenoids. Diacetylenic carotenoids, such as alloxanthin, 7,8,7′,8′-tetradehydroastaxanthin, 4-ketoalloxanthin, and 4′-hydroxy-4-ketoalloxanthin, comprised 13.3% of the total carotenoids.

### 2.3. Carotenoids of P. doliiformis

The carotenoid content and composition of the sea angel *P. doliiformis* are shown in Table 1. *P. doliiformis* contained 159.8 μg/g wet weight carotenoid in the body. This was about three times higher than that of *C. limacina*. It was uncertain why *P. doliiformis* accumulated carotenoids three times higher than *C. limacina*. *P. doliiformis* showed more bright red color than *C. limacina*. This might reflect difference of species. The carotenoid composition of *P. doliiformis* was similar to that of *C. limacina*. Pectenolone (30.5%) was found to be a major component, followed by β-cryptoxanthin (12.8%) and β-carotene (10.2%). The monoacetylenic carotenoid diatoxanthin and its oxidative metabolites, 7,8-didehydroastaxanthin, pectinolone, pectenol A, pectenol B, and 4′-hydroxypectenolone, comprised with 25.9% of the total carotenoids. Diacetylenic carotenoids, alloxanthin, 7,8,7′,8′-tetradehydroastaxanthin, 4-ketoalloxanthin, and 4′-hydroxy-4-ketoalloxanthin, comprised 13.3% of the total carotenoids.

**Table 1:** Carotenoids content and composition of *L. helicina*, *C. limacina*, and *P. doliiformis*.

	*L. helicina*	*C. limacina*	*P. doliiformis*
**Carotenoid content (μg/g wet weight)**	21.0	47.0	159.8
**(μg/specimen)**	0.70	0.75	2.68
**Composition**	**%**	**%**	**%**
β-Carotene (1)	32.2	27.1	10.2
Echinenone (2)		9.2	6.4
Canthaxanthin (3)			3.3
β-Cryptoxanthin (4)	10.4	13.1	12.8
Zeaxanthin (5)	24.2	1.1	1.2
Adonixanthin (6)		9.1	1.4
Idoxanthin (7)			2.5
(3*S*,3′*S*)-Astaxanthin (8)		1.1	5.5
Diatoxanthin (9)	11.1	3.5	3.6
Pectenol A (10)		1.2	2.2
Pectenol B (11)		1.2	2.2
Pectenolone (12)		9.2	30.5
4′-Hydroxypectenolone (13)		4.2	2.5
7,8-Didehydroastaxanthin (14)		4.5	6.4
Alloxanthin (15)	6.4	2.1	1.1
4-Ketoalloxanthin (16)		3.5	2.3
4′-Hydroxy-4-ketoalloxanthin (17)		3.2	2.5
7,8,7′,8′-Tetradehydroastaxanthin (18)		4.5	1.2
Diadinoxanthin (19)	2.4		
Fucoxanthin (20)	5.2		
Others	8.1	2.2	2.2

### 2.4. Carotenoids of the Chum Salmon O. keta

The carotenoid content and composition of flesh of the chum salmon *O. keta*, collected in Monbetsu bay, are shown in Table 2. Acetylenic carotenoids, pectenolone and 7,8-didehydroastaxanthin, were found in *O. keta* as minor carotenoids, along with astaxanthin.

**Table 2:** Carotenoids content and composition of flesh of the chum salmon *O. keta* collected in Monbetsu bay.

Carotenoids Content and Composition of Flesh of the Chum Salmon *O. keta*	
**Carotenoid content (μg/g wet weight)**	0.89
**Composition**	**%**
Astaxanthin *	83.5
9-*cis*-Astaxanthin *	5.1
13-*cis*-Astaxanthin *	2.5
7,8-Didehydroastaxanthin	0.5
Adonixanthin	1.1
Pectenolone	2.5
Others	4.8

* Astaxanthin consisted of three optical isomers (3*R*,3′*R*),(3*R*,3′*S*), and (3*S*,3′*S*) at the ratio of 82:2:16.

## 3. Discussion

**Figure 3:**
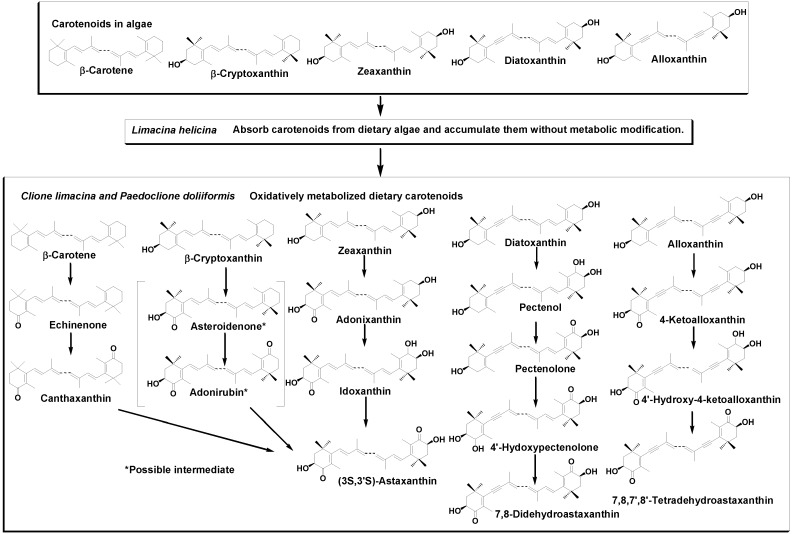
Accumulation and metabolic pathways of carotenoids that originated from phytoplankton in the sea angels *C. limacina* and *P. doliiformis*.

It has been reported that animals do not synthesize carotenoids *de novo*, and so those found in animals are either directly accumulated from food or partly modified through metabolic reactions [6,7,8]. *L. helicina* is a herbivorous animal that feeds on micro algae such as diatoms and dinoflagellates [2]. Sea angels, *C. limacina* and *P. doliiformis* are carnivorous animals that exclusively feed on the small mollusk *L. helicina* [1]. Therefore, carotenoids produced by micro algae are made available to sea angels through *L. helicina* in the food chain. As shown in Table 1, β-carotene, zeaxanthin, diatoxanthin, and β-cryptoxanthin were found to be major carotenoids along with alloxanthin, fucoxanthin, and diadinoxanthin in *L. helicina*. They are characteristic carotenoids in diatoms and microalgae belonging to Cyanophyceae, Rhodophyceae, *etc.* [5,6]. The results indicate that *L. helicina* directly absorbs carotenoids from dietary algae and accumulates them without metabolic modification. On the other hand, keto-carotenoids such as pectenolone, 7,8-didehydroastaxanthin, 4-ketoalloxanthin, and echinenone were found to be major components in sea angels. The results clearly indicate that sea angels oxidatively metabolize ingested carotenoids from *L. helicina*. So, β-carotene was oxidatively converted to astaxanthin via echinenone and canthaxanthin. β-Cryptoxanthin was also metabolized to astaxanthin via asteroidenone and adonirubin, as shown in Figure 3. There are three optical isomers of astaxanthin in nature. However, sea angels contain only one (3*S*,3*′S*) isomer. This shows that hydroxylation at C-3 and/or C-3′ of 4-keto and/or 4′-keto β-end group of carotenoid in sea angels is stereo-selective to form (3*S*,3*′S*)-astaxanthin. This stereo-selective hydroxylation has also been reported in other snails: *Fushinus perplexus*, *F. perplexus ferrugineus*, *F. forceps* [11,12], *Cipangopaludina chinensis laeta*, *Semisulcospia libertina* [13], and *Pomacea canaliculata* [14].

Sea angels also introduced a carbonyl group at C-4 and/or C-4′ in the 3-hydroxy- and/or 3′-hydroxy-β-end group. Namely, zeaxanthin was metabolized to astaxanthin via adonixanthin and idoxanthin. Similarly, an acetylenic carotenoid, diatoxanthin, was metabolized to 7,8-didehydroastaxanthin via pectenol, pectenolone, and 4′-hydroxypectenolone. Alloxanthin was also oxidatively metabolized to 7,8,7′,8′-tetradehydroastaxanthin via 4′-hydroxy-4-ketoalloxanthin, and 4-ketoalloxanthin, as shown in Figure 3. By introducing a carbonyl group at C-4 and/or C-4′ in the 3-hydroxy- and/or 3′-hydroxy-β-end group, carotenoids changed their color from yellow to red. Therefore, the red color of the gonads of sea angels is due to the presence of keto-carotenoids such as pectenolone, 7,8-didehydroastaxanthin, and 7,8,7′,8′-tetradehydroastaxanthin. Epoxy carotenoids, diadinoxanthin and fucoxanthin, which are present in *L. helicina*, were not found in sea angels. It is suggested that sea angels cannot absorb epoxy carotenoids.

Chum salmon, *O. keta*, feeds not only on micro crustaceans but also on sea angels [3,4,5]. Astaxanthin, which consists of three optical isomers, was found to be a major carotenoid, along with the acetylenic carotenoids pectenolone and 7,8-didehydroastaxanthin, in *O. keta*. It is well-known that astaxanthin in crustaceans such as krill also consists of three optical isomers [6,7,8,15]. Therefore it is clear that astaxanthin in salmon originates from crustaceans. On the other hand, the acetylenic carotenoids pectenolone and 7,8-didehydroastaxanthin were not found in these crustaceans [6,7,8,15]. So, they are suggested to originate from sea angels.

It has been reported that marine animals accumulate carotenoids in their gonads, such as astaxanthin in salmon, pectenolone in scallops, and echinenone in sea urchins and that carotenoids are essential for reproduction in marine animals [8]. For example, astaxanthin supplementation in cultured salmon and red sea bream increased ovary development, fertilization, hatching, and larval growth [16]. In the case of sea urchins, supplementation with β-carotene, which was metabolized to echinenone, also increased reproduction and the survival of larvae [17].

As described above, sea angels converted dietary carotenoids to corresponding keto-carotenoids by introducing a carobonyl group and accumulated these keto-carotenoids in their gonads. Several investigators have reported that introducing a carobonyl group at C-4 and/or C-4′ of the β-end group of carotenoids enhanced their antioxidant effects, such as the quenching of singlet oxygen (^1^O_2_), inhibiting lipid peroxidation, and protection from photo-oxidation [18,19,20,21]. As well as astaxanthin, pectenolone, an oxidative metabolite of diatoxanthin, showed excellent antioxidative activity by inhibiting lipid peroxidation [22] and quenching singlet oxygen (^1^O_2_). Therefore, keto-carotenoids such as pectenolone may contribute to protection against oxidative stress and promote the reproduction of sea angels through antioxidative activity.

## 4. Experimental Section

### 4.1. General

The UV-visible (UV-VIS) spectra were recorded with a Hitachi U-2001 (Hitachi High-Technologies Corporation, Tokyo, Japan) in diethyl ether (Et_2_O). The positive ion electro spray ionization time of flight mass (ESI-TOF MS) spectra were recorded using a Waters Xevo G2S Q TOF mass spectrometer (Waters Corporation, Milford, CT, USA). The ^1^H-NMR (500 MHz) spectra were measured with a Varian UNITY INOVA 500 spectrometer (Agilent Technologies, Santa Clara, CA, USA) in CDCl_3_ with TMS as an internal standard. HPLC was performed on a Shimadzu LC-6AD with a Shimadzu SPD-6AV spectrophotometer (Shimadzu Corporation, Kyoto, Japan) set at 470 nm. The column used was a 250 × 10 mm i.d., 10 μm Cosmosil 5C18-II (Nacalai Tesque, Kyoto, Japan) with acetone:hexane (3:7, v/v) at a flow rate of 1.0 mL/min, run time of 60 min. The optical purity of astaxanthin was analyzed by chiral HPLC using a 300 × 8 mm i.d., 5 μm Sumichiral OA-2000 (Sumitomo Chemicals, Osaka, Japan) with *n*-hexane/CHCl_3_/ethanol (48:16:0.8, v/v) at a flow rate of 1.0 mL/min [23].

### 4.2. Animal Specimens

The sea angel *C. limacina* (30 specimens, 464 mg wet weight) was collected at Monbetsu bay, Monbetsu City, Hokkaido, Japan in December 2011. Another sea angel, *P. doliiformis* (60 specimens, 1041 mg wet weight), was also collected at Monbetsu bay in April 2013. The small sea snail *L. helicina* (6 specimens, 200 mg wet weight) was collected at Monbetsu bay in May 2013. Chum salmon, *O. keta* (3 specimens, five to six years of age), was collected at Monbetsu in September 2013.

### 4.3. Analysis of Carotenoids

The extraction and identification of carotenoids were carried out according to our routine methods [24]. Carotenoids were extracted from living or fresh animal specimens with acetone. The acetone extract was translated to an ether-hexane (1:1) layer by the addition of water. The total carotenoid contents were calculated employing an extinction coefficient of 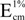 = 2100 [25] at λ max. The ether-hexane solution was evaporated. The residue was subjected to HPLC on silica gel. Carotenoid compositions were estimated by the peak area of the HPLC on silica gel with acetone–hexane (2:8)–(4:6) monitored at 450 nm.

Individual carotenoids were identified by retention time in HPLC, UV-vis (ether), ESI-TOF MS, and ^1^H NMR (500 MHz, CDCl_3_) in the case of pecetenolone.

### 4.4. Identification of Carotenoids

β-Carotene (**1**). ESI-TOF MS: *m/z* 536.4372 [M]^+^ (calcd for C_40_H_56_, 536.4382); UV-VIS: 425, 449, 475 nm.

Echinenone (**2**). ESI-TOF MS: *m/z* 551.4271 [M + H]^+^ (calcd for C_40_H_53_O, 551.4253); UV-VIS: 460 nm.

Canthaxanthin (**3**). ESI-TOF MS: *m/z* 565.4044 [M + H]^+^ (calcd for C_40_H_53_O_2_, 565.4046); UV-VIS 470 nm.

β-Cryptoxanthin (**4**). ESI-TOF MS: *m/z* 553.4511 [M + H]^+^ (calcd for C_40_H_53_O, 553.4409); UV-VIS: (425), 450, 475 nm.

Zeaxanthin (**5**). ESI-TOF MS: *m/z* 569.4353 [M + H]^+^ (calcd for C_40_H_57_O_2_,569.4359); UV-VIS: (425) 450, 475 nm.

Adonixanthin (**6**). ESI-TOF MS: *m/z* 583.4139 [M + H]^+^ (calcd for C_40_H_55_O_3_, 583.4151); UV-VIS 460 nm.

Idoxanthin (**7**). ESI-TOF MS: *m/z* 599.4090 [M + H]^+^ (calcd for C_40_H_55_O_4_, 599.4100); UV-VIS 460 nm.

Astaxanthin (**8**). ESI-TOF MS: *m/z* 597.3942 [M + H]^+^ (calcd for C_40_H_53_O_4_, 597.3944); UV-VIS 472 nm, Chiral HPLC [13] revealed that astaxanthin fraction in sea angels was consisted of only (3*S*,3′*S*) optical isomers.

Diatoxanthin (**9**). ESI-TOF MS: *m/z* 567.4225 [M + H]^+^ (calcd for C_40_H_55_O_2_, 567.4202); UV-VIS: (426), 451, 478 nm.

Pectenol A (**10**). ESI-TOF MS: *m/z* 583.4173 [M + H]^+^ (calcd for C_40_H_55_O_3_, 583.4152); UV-VIS: (426), 451 478 nm.

Pectenol B (**11**). ESI-TOF MS: *m/z* 583.4170 [M + H]^+^ (calcd for C_40_H_55_O_3_, 583.4152); UV-VIS: (426), 451, 478 nm.

Pectenolone (**12**). ESI-TOF MS: *m/z* 581.3983 [M + H]^+^ (calcd for C_40_H_53_O_3_, 581.3995); UV-VIS: 460 nm; ^1^H-NMR (CDCl_3_, 500 MHz) δ 1.15 (H_3_-16′, s), 1.20 (H_3_-17′, s), 1.21 (H_3_-17, s), 1.32 (H_3_-16, s), 1.45 (H-2′β, dd, *J* = 12, 11), 1.82 (H-2β, d, *J* = 13, 13), 1.84 (H-2′α, ddd, *J* = 12, 4, 1.5), 1.92 (H_3_-19′, s), 1.95 (H_3_-19, s), 2.07 (H-2′β, dd, *J* = 18, 10), 2.15 (H-2α, dd, *J* = 13, 6), 2.43 (H-4′α, ddd, *J* = 18, 6, 1.5), 3.68 (OH-3, d, *J* = 2), 3.99 (H-3′, m), 4.32 (H-3, ddd, *J* = 13, 6, 2), 6.22 (H-7, d, *J* = 16), 6,28 (H-14′, d, *J* = 11), 6.30 (H-10, d, *J* = 11), 6.30 (H-14, d, *J* = 11), 6.36 (H-12′, d, *J* = 15), 6.43 (H-8, d, *J* = 16), 6.45 (H-12, d, *J* = 15), 6.45 (H-10′, d, *J* = 11), 6.53 (H-11′, dd, *J* = 15, 11), 6.63 (H-15 and H-15′, m), 6.65 (H-11, dd, *J* = 15, 11).

4′-Hydroxypectenolone (**13**). ESI-TOF MS: *m/z* 597.3942 [M + H]^+^ (calcd for C_40_H_53_O_4_, 597.3944); UV-VIS: 460 nm.

7,8-Didehydroastaxanthin (**14**). ESI-TOF MS: *m/z* 595.3789 [M + H]^+^ (calcd for C_40_H_51_O_4_, 595.3787); UV-VIS: 474 nm.

Alloxanthin (**15**). ESI-TOF MS: *m/z* 565.4028 [M + H]^+^ (calcd for C_40_H_53_O_2_, 565.4046); UV-VIS: (426), 451 478 nm.

4-Ketoalloxanthin (**16**). ESI-TOF MS: *m/z* 579.3851 [M + H]^+^ (calcd for C_40_H_51_O_3_, 579.3838); UV-VIS: 460 nm.

4′-Hydroxy-4-Ketoalloxanthin (**17**). ESI-TOF MS: *m/z* 595.3801 [M + H]^+^ (calcd for C_40_H_51_O_4_,595.3787); UV-VIS: 469 nm.

7,8,7′,8′-Tetradehydroastaxanthin (**18**). ESI-TOF MS: *m/z* 593.3649 [M + H]^+^ (calcd for C_40_H_49_O_4_,593.3631); UV-VIS: 476 nm.

Diadinoxanthin (**19**). ESI-TOF MS: *m/z* 583.4173 [M + H]^+^ (calcd for C_40_H_55_O_3_, 583.4151); UV-VIS: 420, 433, 470 nm. 

Fucoxanthin (**20**). ESI-TOF MS: *m/z* 659.4333 [M + H]^+^ (calcd for C_42_H_59_O_6_,659.4312); UV-VIS: 445, 470 nm.

### 4.5. ^1^O_2_ Quenching Activity of Carotenoids

Quenching activity of ^1^O_2_ was measured according to the method described in the literature [26]. ^1^O_2_ quenching activities (IC_50_ values) of pectenolone and astaxanthin were 7.9 and 6.5 μM, respectively.

## 5. Conclusions

Carotenoids originating from phytoplankton are accumulated in the sea angels, *C. limacina* and *P. doliiformis*, through eating the herbivorous sea snail, *L. helicina*, in the food chain. In sea angels, dietary carotenoids were oxidatively metabolized, as shown in Figure 3. Sea angels mainly accumulate carotenoids in their gonads. Carotenoids in the gonads of sea angels might protect against oxidative stress and enhance reproduction. Furthermore, carotenoids in sea angels can then be found in salmon through the food chain.
